# An investigation of somatosensory profiles in work related upper limb disorders: a case-control observational study protocol

**DOI:** 10.1186/1471-2474-11-22

**Published:** 2010-01-30

**Authors:** Niamh Moloney, Toby Hall, Catherine Doody

**Affiliations:** 1 UCD School of Public Health, Physiotherapy and Population Science, University College Dublin, Belfield, Dublin 4, Ireland; 2 School of Physiotherapy, Curtin University of Technology, G.P.O. Box U1987, Perth, WA 6845, Australia; 3 Manual Concepts, P.O. Box 1236, Booragoon, WA 6954, Australia

## Abstract

**Background:**

Work related upper limb disorders constitute 45% of all occupational diseases and are a significant public health problem. A subgroup, non specific arm pain (NSAP), remains elusive in terms of understanding its pathophysiological mechanisms with its diagnosis based on the *absence *of specific clinical findings. One commonly proposed theory is that a neural tissue disorder is the primary dysfunction in NSAP and findings from previous studies lend some support to this theory. However, it is not clear if changes identified are simply a consequence of ongoing pain rather than due to specific neural changes. The presence of neuropathic pain has been investigated in several other musculoskeletal conditions but currently, there is no specific diagnostic tool or gold standard which permits an unequivocal diagnosis of neuropathic pain. The purpose of this study is to further describe the somatosensory profiles in patients with NSAP and to compare these profiles to a group of patients with MRI confirmed cervical radiculopathy who have been previously classified as having neuropathic pain.

**Methods/Design:**

Three groups of participants will be investigated: Groups 1 and 2 will be office workers with either NSAP or cervical radiculopathy and Group 3 will be a control group of non office workers without upper limb pain. Participants will undergo a clinical assessment, pain questionnaires (LANSS, Short Form McGill, DASH and TSK) and quantitative sensory testing comprising thermal detection and pain thresholds, vibration thresholds and pressure pain thresholds.

**Discussion:**

The spectrum of clinically suspected neuropathic pain ranges from more obvious conditions such as trigeminal neuralgia to those with vague signs of nerve disorder such as NSAP. A thorough description of the somatosensory profiles of NSAP patients and a comparison with a more defined group of patients with evidence of neuropathic pain will help in the understanding of underlying neurophysiology in NSAP and may influence future classification and intervention studies relating to this condition.

## Background

Work related upper limb disorders (WRULD) (often called repetitive strain injury) are a significant public health problem, estimated to constitute 45% of all occupational diseases [1]. Non specific arm pain (NSAP) constitutes a subgroup of WRULD [2-5] and has been defined as diffuse pain in the forearm (which can also involve the neck, arm, wrist and hand) in the absence of evidence of a specific upper limb disorder [2-6].

### Aetiology of NSAP

NSAP is a vague clinical entity, the pathophysiological mechanisms of which remain unclear. While it is quite feasible that pain in NSAP is nociceptive in origin e.g. from muscle tissue, an underlying dysfunction of the nervous system has been proposed [7-9]. A mechanisms based approach to understanding pain has been advocated [10] but currently, there are no gold standards or valid or reliable methods to diagnose underlying neurophysiological pain mechanisms. It is proposed that the use of a pain mechanisms classification system would be valuable in identifying sub-groups of patients who are most likely to require different treatment strategies [10]. There is some evidence to suggest that patients treated according to symptom specific classification systems have superior outcomes to those who are not [11].

A number of algorithms for assessing the presence of neuropathic pain exist. They include the use of pain questionnaires and combinations of pain descriptors and clinical testing [12-14]. Treede et al's [14] proposed algorithm for the assessment of neuropathic pain involves the development of a working hypothesis of "possible" neuropathic pain based on clinical information, pain mapping and history relating to the onset of pain. If further evidence by way of clinical examination, neurophysiological tests and laboratory tests such as MRI can be yielded, then patients can be categorised further as "probable" or "definite" neuropathic pain. However, this model requires further testing in terms of reliability and validity. In addition, there are a number of pain questionnaires for the classification of neuropathic pain (e.g. Leeds Assessment for Neuropathic Signs and Symptoms (LANSS), Neuropathic Pain Questionnaire, pain DETECT). Again, all of these methods require further validation before their use as a diagnostic tool can be advocated.

Another means of assessing pain mechanisms is the use of quantitative sensory testing (QST). QST is useful in quantifying mechanical and thermal allodynia and hyperalgesia and may help to characterise painful neuropathic syndromes as well as clarifying some of their pathophysiological mechanisms [15]. However, QST alone cannot conclusively determine the presence of neuropathic pain.

### Evidence for the presence of Neuropathic Pain in NSAP

In relation to the clinical presentation in NSAP, many researchers have reported presenting symptoms which are consistent with those of neuropathic pain, for example, paraesthesia, dysaesthesia, weakness, burning pain, cramping, slowing of fine movements and allodynia [5-9,16,17]. This has led to the initial hypothesis that NSAP may have an underlying neuropathic pain component. Clinical studies have identified the presence of mechanical hyperalgesia involving the peripheral nerve trunks in this population [16-19]. Peripheral nerve trunk mechanical hyperalgesia is tested using upper limb neurodynamic tests and these have been identified as useful indicators of central nervous system hyperexcitability [20]. They may also be reflective of localised peripheral nerve inflammation [21] or indicative of peripheral nerve sensitisation [12]. Smart et al., (2009) [13] have found neural tissue provocation tests for the lower limb (straight leg raise) a useful indicator of peripheral nerve sensitisation while Scholz et al., (2009)[22] have also identified the straight leg raise test within a battery of tests as useful for the differentiation of neuropathic pain mechanisms in LBP. However, the correlation of neurodynamic tests with qualitative measures of pain and quantitative measures of nerve function or indeed the presence of neuropathic pain is unknown in NSAP.

Various QST measures have been used to investigate the somatosensory profiles of patients with NSAP; however the results of these studies reveal conflicting evidence. Greening et al., [23,24] have identified changes in flare response, vasoconstriction and vibration in office workers with NSAP when compared with office workers without pain or non office workers. Their findings in relation to vibration are supported by some researchers [25-28], but not others [29,30].

### QST findings in musculoskeletal disorders

While pressure pain thresholds or thermal QST have not been specifically investigated in NSAP, studies of other working populations have yielded mixed results. Increased sensitivity to pressure has been found in the shoulder and neck muscles of butchers [31], secretaries [32] and manufacturing workers [33]. Contrary to this, Johnston et al., [34] found office workers with neck pain showed no difference in pressure pain thresholds (PPT) over the neck muscles in comparison to controls but they did have lower PPT at median nerve and tibialis anterior sites. Increased sensitivity to pressure has also been found in whiplash and cervical radiculopathy patients [35,36]. In relation to thermal QST, Johnston et al., [34] found that office workers with mild neck pain and disability had decreased heat pain thresholds and were more sensitive to cold pain than controls or office workers without pain. Furthermore, alterations in thermal detection thresholds have been found in the upper limbs of patients with whiplash and cervical radiculopathy [37]. One interesting finding in this study by Chien et al. is the presence of non-symptomatic to symptomatic side differences in the cervical radiculopathy group which did not exist in the whiplash group. Whilst further investigations are warranted to elucidate the significance of this finding, it may reflect a difference between neuropathic versus non-neuropathic pain presentations.

Recent investigations into work related neck pain [34]; diffuse upper limb pain [28], low back pain [38], whiplash [20,36], patellofemoral pain syndrome [39], fibromyalgia and complex regional pain syndrome [40] have shown that these conditions may have a neuropathic pain component. However, many of these studies show positive findings on both the asymptomatic and symptomatic sides, indicating that change may just be reflective of alterations in somatosensory processing associated with pain i.e. central plasticity from nociceptive or inflammatory pain as opposed to true neuropathic pain [14].

Clearly, there is much debate as to whether NSAP, which appears to have features of neuropathic pain, is indeed a neuropathic disorder. Furthermore, there are currently no gold standards with which to classify pain as definitively neuropathic. The purpose of this study is to comprehensively describe the somatosensory profiles in patients with NSAP and to compare them to somatosensory profiles in asymptomatic subjects and subjects with cervical radiculopathy. It is hypothesised that some of the NSAP group may demonstrate a similar presentation to that of cervical radiculopathy, indicating a probable neuropathic pain presentation.

## Methods/Design

### Objectives

The objectives of this study are to:

• Describe the somatosensory profiles of subjects with NSAP in terms of neurological examination, qualitative measures of pain, neural tissue provocation tests and QST.

• Describe impairment and disability associated with NSAP by means of the Disabilities of the Arm, Shoulder and Hand (DASH) and SF-36 questionnaires.

• Investigate for correlations between clinical examination findings, QST profiles and questionnaire derived pain profiles in subjects with NSAP.

• Compare somatosensory profiles of subjects with NSAP to those with cervical radiculopathy and asymptomatic controls.

### Study design

A case-control observational design will be used to assess quantitative and qualitative sensory features as well as pain profiles in two patient groups and a control group.

### Ethical Approval

The study was approved by the Human Research Ethics Committee - Life Sciences in University College Dublin on January 23rd 2009.

### Participants

One hundred and fifty consenting male and female subjects, aged 18-65 years will be recruited comprising three equal groups of (1) Patients with NSAP, (2) Patients with cervical radiculopathy and (3) Age and gender matched controls.

#### • Groups 1 and 2: NSAP and Cervical Radiculopathy subjects

##### Inclusion criteria

All participants from patient groups will be office workers who have significant unilateral upper limb pain as defined by a numerical rating score of ≥ 3/10 [31,41], who spend more than 40% of their working week using desktop equipment [24] and have been in their current employment or similar (i.e. involving office work/keyboard operation) for at least two years [34]. More specific information regarding diagnostic criteria for each patient group is outlined in the next section. Patients will be recruited from local orthopaedic outpatient and occupational health departments of Dublin hospitals and physiotherapy practices and also from the staff and student population of University College Dublin.

#### • Group 3: Control Subjects

##### Inclusion criteria

Fifty age and gender matched subjects will be included if they have no history of significant neck, scapular or shoulder pain over the past 12 months (significant pain is defined as pain ≥ 3/10 on a visual analogue scale [31,41] and do not use desktop equipment for more than 40% of their working week [24]. The control group will be recruited from staff/students at University College Dublin, St Vincent's University Hospital staff and the local community.

### Exclusion criteria for all participants

Subjects will be excluded if they are seeking compensation for their injury or if they have any of the following: bilateral upper limb pain, neurological disorders, generalized musculoskeletal disorders e.g. rheumatoid arthritis or fibromyalgia, a history of low back pain and/or low back related leg pain over the previous 6 months, a history of migraine over the previous 6 months, previous surgery or trauma to the upper quadrant, diabetes or endocrine disorders, epilepsy or any psychiatric disorders. Participants taking anti-epileptic or anti-depressant medication, or who are undergoing chemotherapy/radiotherapy will also be excluded.

### Group Selection

#### Screening and Allocation to study groups

**Figure 1:**
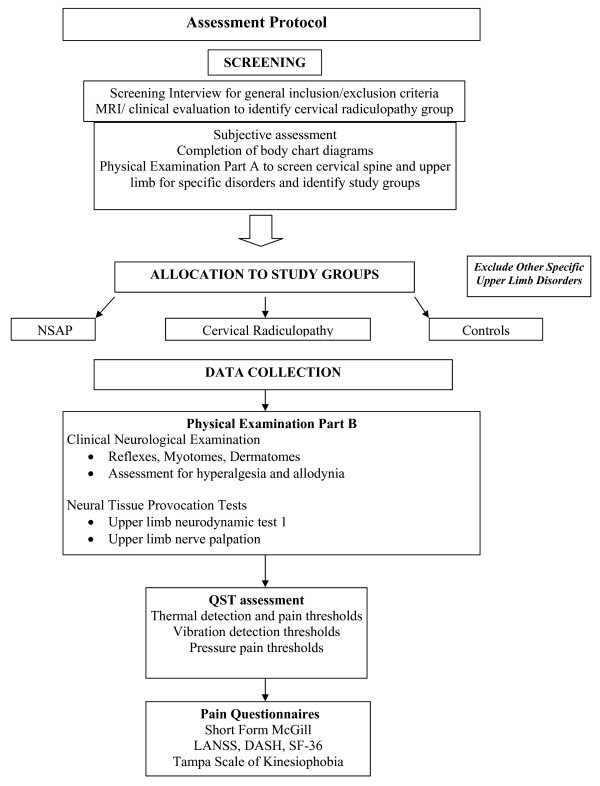
**Study Protocol**.

Participants will be interviewed and screened for general inclusion and exclusion criteria (Figure 1: Study Protocol). Participants with cervical radiculopathy will be recruited from a local spinal surgery service and identified by the presence of radicular pain in the upper limb, positive upper limb neurodynamic tests, Spurling's test, and Valsalva manoeuver as well as MRI confirmation of nerve compression [42,43]. The MRI scans of all patients will be reviewed by a radiologist and those patients with evidence of intervertebral disc protrusion and associated nerve root compression will be identified. Nerve root compression will be classified as absent, minimal, moderate or severe [44].

All other participants will be screened by means of a subjective examination, pain area mapping and a clinical orthopaedic examination to identify participants with NSAP and to exclude patients with all other specific upper limb disorders according to classification criteria outlined by Boocock et al, [2]. The classification criteria for NSAP are listed in Table 1, with classification criteria for specific upper limb disorders listed in Additional file 1.

**Table 1:** Classification criteria for NSAP

*DEFINITION NSAP*	*SYMPTOMS*	*SIGNS*
Pain in the arm in the absence of a specific diagnosis or pathology	Pain in the forearm and failure to meet the diagnostic criteria of specific disorders *Symptoms may include:* Loss of function Weakness in arms and hands Cramp Muscle tenderness Allodynia Slowing of fine movements Clumsiness	Absence of signs of a specific pathology that correlates with area of pain.

Boocock et al., 2009

### Data Collection

General information regarding subject health, work status, and medication use will be obtained. Information regarding the work practices of each individual will also be collected, including how many hours they spend using desktop, laptop and mouse equipment.

### Physical Examination Part B

All measures will be performed by the principal investigator. Whilst independent screening and allocation to groups would be preferable, this is not feasible within the current study and possible resultant bias is acknowledged by the authors.

#### Neurological examination

A bedside neurological evaluation of the upper limb will be carried out on all subjects, including assessment of upper limb reflexes, myotomes and sensation.

##### • Deep tendon reflexes

Deep tendon reflexes will be assessed for Biceps (C5/6), Brachioradialis (C5/6), Pronator Teres (C**6**/7) and Triceps (C**7**/8) [45,46]. As any grade of reflex can be considered normal, reflexes will be rated normal or abnormal by comparison to the other side as it is considered that asymmetry is the most helpful assessment [47]. If reflexes are found to be abnormal, they will be further categorised as absent/diminished or increased [48]. Interrater reliability using this grading system has been found to be good for the biceps reflex [48].

##### • Myotomes

Myotomes will be assessed by testing the strength of muscles corresponding to each nerve root according to previously published guidelines [49-52]. Interrater reliability for manual muscle tests ranges from κ = 0.53- 0.83 when measuring muscle strength as normal or reduced [48]

##### • Dermatomes

Sensation will be assessed for detection of light touch (using cotton wool) and sharp-blunt discrimination (using a pin prick). Stimuli will be applied circumferentially repeatedly around the participant's upper limbs so that all dermatomes will be assessed, with both limbs assessed for comparison. Each finger will be assessed separately [48]. When a difference is detected the area will be assessed in more detail and a map of the area of altered sensation will be recorded on a body chart to determine if the area follows a dermatomal pattern, is consistent with a particular peripheral nerve or if it corresponds to somatic referred pain or trigger point referral patterns [53,54]. Each sensory level will be graded as reduced, normal or increased [48]. Interrater reliability for assessment of dermatomes has been found to be moderate [48,50]. In addition, areas of heightened sensation will be assessed for signs of allodynia or hyperalgesia [15].

#### Neural Tissue Provocation Tests

Mechanosensitivity of upper limb neural tissue will be assessed using upper limb neurodynamic tests and nerve palpation.

##### • Neurodynamic Test

Longitudinal tests of the brachial plexus and peripheral nerves of the upper limb called the upper limb neurodynamic tests (ULNT) have been shown to reliably detect neural tissue mechanosensitivity [55]. The ULNT^1 ^has received the most research investigation and will be used in this study [16,35,56-60]. Interrater reliability of the ULNT^1 ^has been shown to be moderate (κ = 0.54) [43] and good (κ = 0.73) [48].

With the participant supine the test will be performed in the following sequence: gentle scapular depression, shoulder abduction, forearm supination combined with wrist and finger extension, shoulder external rotation and elbow extension [37]. Participants will be asked to indicate to the examiner during elbow extension when they first perceive pain anywhere along the tested arm. The angle of elbow extension will then be measured using a standard goniometer aligned along the mid-humeral shaft, medial epicondyle and ulnar styloid [61] and the participant will be asked to indicate their pain experienced during the test using the numerical rating scale [37].

##### • Nerve Palpation

The median, radial and ulnar nerves will be digitally palpated to assess for mechanosensitivity [62]. Nerve palpation has been used in previous studies and has shown to be reproducible [50] and moderately reliable [48]. Moderate digital pressure will be applied to each nerve at the following sites: the median nerve in the cubital fossa medial to and immediately adjacent to the biceps tendon; the ulnar nerve in the groove between the median epicondyle and the olecranon, the radial nerve in the upper arm where it passes through the intermuscular septum between the medial and lateral heads of triceps [63-65]. A test will be deemed positive if pain or symptoms are elicited [48].

### Quantitative Sensory Testing

A familiarisation trial will be performed on the brachioradialis muscle for all measures of QST prior to testing [35]. The sequence of examination will be randomised using a random number generator in order to control for the effects of fatigue.

#### • Thermal Testing

All tests will be performed using a NeuroSensory Analyser (TSA 2001 II Medoc, Israel). The Peltier thermode (16 × 16 mm) will be attached directly over areas in the hand innervated by C6 (dorsum of the first metacarpal), C7 (dorsum of the second metacarpal) and C8 (dorsum of the fifth metacarpal). Tibialis anterior will be used as a reference point [35]. The temperature of the NeuroSensory Analyser will be preset to a baseline of 30 degrees Celsius and increased or decreased at a rate of 1 degree per second. Thermal detection (Warm, Cold) and pain (Heat, Cold) thresholds will be measured using the method of limits [35]. Standardised instructions will be read out to the participants prior to testing. In the case of warm and cold detection thresholds participants will be asked to indicate when they notice the temperature of the thermode changing from neutral to warm/cool and in the case of heat and cold pain thresholds participants will be asked to indicate when they notice the heat or cold becoming painful. Participants will be asked to press a patient control switch at each point. Participants will be advised that this is not a measure of pain tolerance. All measures will be taken in triplicate with the mean values used for analysis [66]. Thermal QST measures have been shown to demonstrate reasonable test-retest reliability [41,66,67].

#### • Vibration thresholds

A Vibrameter (VSA 3000 II 2001 Medoc, Israel) will be used to measure vibration perception thresholds. Readings will be taken over the areas of the hand innervated homonymously by C6 (palmar aspect of the first metacarpal), C7 (palmar aspect of the second metacarpal) and C8 dermatomes (dorsum of the fifth metacarpal). Triplicate recordings will be taken at each site and the mean values used for analysis [35]. Test - retest reliability and [68-72] inter - rater reliability [69] has been shown to be consistently good for vibration measurements.

#### • Pressure Pain Thresholds

Pressure pain thresholds (PPT) will be determined using a hand held pressure algometer with a probe size of 1 cm2 (Somedic AB, Farsta, Sweden) and an application rate of 40 kPa/s. PPT will then be recorded bilaterally over the median, ulnar and radial nerves, the sites of which will correspond to the nerve palpation sites outlined above [65]. All sites will be marked with a skin marker to ensure accuracy [73]. The participant will be instructed to press a patient control switch when the sensation under the probe changes from one of pressure to one of pressure and pain. All measures will be taken in triplicate and the mean value was used for analysis [35]. PPT measures have been repeatedly shown to demonstrate good intra and inter - rater reliability both in general [74-78] and in sites specific to the upper limb nerves [65]

### Pain Assessment

Participants from both patient groups will be asked to complete a detailed assessment of pain intensity and quality. A clinically administered LANSS questionnaire will be used for specific identification of neuropathic pain. The LANSS has been tested and validated in several settings [79-81]. A Short Form McGill Pain Questionnaire will be administered [82], which has been validated for the measurement of sensory and affective dimensions of pain [83]. The McGill includes a visual analogue scale and present pain index [82,84]. A Tampa Scale for Kinesiophobia will be used as this has been shown to be a reliable assessment tool for chronic pain [85-87,82-84] while the DASH [88,89] and SF-36 will be used as measures of disability and functional impairment in these patient groups. Furthermore, three proposed algorithms, which are currently under review by other researchers, will be used as aids in differentiation of pain mechanisms for all symptomatic subjects [12-14].

### Data Management

Participant data will be coded to ensure anonymity of participants and stored at a central database at the School of Physiotherapy and Performance Science, University College Dublin, Ireland.

### Sample size/power calculation

Power analysis: Sample size was calculated based on mean and standard error vibration threshold data from a study by Greening et al., (2003). A sample of size of 50 patients with NSAP, 50 patients with cervical radiculopathy and 50 matched control subjects will be required to detect a medium effect size (0.5) with 0.8 power and 0.05 two tailed significance level.

### Statistical Analysis

Descriptive statistics will be calculated for all outcome measures. Differences in categorical variables between groups will be analysed using a Chi Square Test. A one way ANOVA with Student-Newman-Keuls test will be used for Post-hoc comparisons for all continuous normally distributed data.

### Study Limitations

• The screening, group allocation and data collection will all be carried out by the principal investigator. Whilst independent screening and group allocation would be preferable, this is not feasible. The authors acknowledge the bias inherent in this.

• NSAP participants could be investigated with MRI and/or nerve conduction studies to rule out any identifiable nerve dysfunction and the authors acknowledge this limitation.

## Discussion

### Scientific significance

WRULD are a significant public health problem and NSAP constitutes a diagnostic category for which our understanding of pathophysiological mechanisms remains unclear. The spectrum of clinically suspected neuropathic pain ranges from more obvious conditions such as trigeminal neuralgia to those with vague signs of nerve disorder such as NSAP. A thorough description of the somatosensory profiles of NSAP patients and a comparison with a more defined group of patients with probable neuropathic pain will help in the understanding if underlying neurophysiology in this condition and may influence future classification and intervention studies relating to NSAP.

## Abbreviations

**WRULD**: Work related upper limb disorders; **NSAP**: Non specific arm pain; **QST**: Quantitative Sensory Testing; **PPT**: Pressure Pain Threshold; **LANSS**: Leeds Assessment of Neuropathic Symptoms and Signs; **ULNT**: Upper limb neurodynamic tests; **DASH **Disabilities of the Arm; Shoulder and Hand; **TSK**: Tampa Scale of Kinesiophobia.

## Competing interests

The authors declare that they have no competing interests.

## Authors' contributions

NM, CD and TH all participated fully in the development of the study protocol. NM drafted this manuscript which was reviewed and critiqued by CD and TH. All authors read and approved the final manuscript.

## Authors' informations

NM is a Musculoskeletal Physiotherapist with an interest in neural tissue disorders. She is currently a PhD student in University College Dublin.

CD is a Lecturer in Physiotherapy at University College Dublin with a special interest in clinical reasoning and musculoskeletal disorders.

TH is a Specialist Musculoskeletal Physiotherapist and Adjunct Senior Teaching Fellow at Curtin University of Technology, Perth, Western Australia. His research interests are neural tissue pain disorders and cervicogenic headache.

## Pre-publication history

The pre-publication history for this paper can be accessed here:

http://www.biomedcentral.com/1471-2474/11/22/prepub

## Supplementary Material

Additional file 1 **Classification Criteria for Work Related Upper Limb Disorders (Boocock 2009)**. Consensus diagnostic criteria for specific upper limb disorders. Click here for file
