# Supplementary material for: An investigation of somatosensory profiles in work related upper limb disorders: a case-control observational study protocol
Source: BMC Musculoskelet Disord. 2010 Jan 30;11:22. doi: 10.1186/1471-2474-11-22 (PMC2825226; doi:10.1186/1471-2474-11-22)
Supplement: Additional file 1 — Classification Criteria for Work Related Upper Limb Disorders (Boocock 2009). Consensus diagnostic criteria for specific upper limb disorders. [file 1471-2474-11-22-S1.DOC]

**Additional file 1.**

**Title: Classification Criteria for Work Related Upper Limb Disorders (Boocock 2009)**

| **CARPAL TUNNEL SYNDROME**  A clinical syndrome caused by compression of the median nerve in carpal tunnel | - Pain/Paraesthesia Median nerve distribution - Nocturnal exacerbation of symptoms - Symptoms present now or on at least 4 days during the last 7 days   Additional Features:   - Relative sparing of dorsum of hand and little finger - No other apparent cause - History of successful steroid injection/surgery - Absence of depression, early morning stiffness, finger joint pain or swelling rest pain | At least 1 of the following positive:   - Positive Tinels test - Positive Phalens test - Motor loss/atrophy of abductor pollicis brevis/ thenar musculature - Probable or classic Hand Pain Diagram - Abnormal Semmes -Weinstein test - Positive compression test - Abnormal nerve conduction studies - Loss of two point discrimination |
| --- | --- | --- |
| **ULNAR NERVE COMPRESSION AT THE ELBOW- CUBITAL TUNNEL SYNDROME** | - At least intermittent pain or paraesthesia in the 4^th^ and/or 5^th^ digits - AND pain in the medial aspect of the elbow - OR pain in the ulnar innervated area of the hand - Symptoms worse at night - Symptoms present now or on at least 4 days during the last 7 days - Decreased sensation in the little finger and ulnar half of the ring finger - Loss of power, grip or dexterity | - Positive elbow flexion/ulnar compression tests - Atrophy or weakness of the ulnar intrinsic muscles of the hand - Pain on palpation of the medial aspect of the elbow - Weakness in abduction of fingers with interphalangeal joins in extension - Clawing contracture of the ring and little fingers –Benediction sign - Wartenberg’s sign - Froments sign - Positive Tinels test |
| **ULNAR NERVE COMPRESSION AT THE WRIST: GUYON CANAL SYNDROME** | - Intermittent paraesthesia in the palmar ulnar nerve distribution of the hand distal to the wrist - OR pain in the ulnar innercated area of the hand, which may radiate to the forearm - Symptoms present now or on at least 4 days during the last 7 days | At least 1 of the following:   - Weakness or atrophy in the ulnar innervated intrinsic hand muscles - Positive Tinel’s test - Positive Reversed Phalen’s test - Positive pressure test over the guyon canal |
| **RADIAL NERVE COMPRESSION: RADIAL TUNNEL SYNDROME** | - Pain in the lateral elbow region or forearm muscle mass of the wrist extensor-supinator - OR weakness on extending the wrist and fingers - Symptoms present now or on at least 4 days during the last 7 days | - Tenderness in the supinator area on palpation over the radial nerve 4-7cm distal to the lateral epicondyle   AND at least 1 of the following positive:   - Weakness on resisted forearm supination - Weakness on resisted middle finger extension |
| **ROTATOR CUFF TENDINOPATHY**  Symptomatic inflammation or degeneration of the tendons of the rotator cuff or biceps | **Rotator Cuff**: Pain in deltoid area, often worse at night, aggravated by shoulder movement  Absence of finger joint pain or swelling, pain in the hand or wrist, hand clumsiness, paraesthesia  **Biceps Tendinopathy**: Anterior shoulder pain | **Rotator Cuff**  Pain on 1 or more active resisted movements: abduction, external rotation or internal rotation   - Painful arc on active abduction/elevation - Limitation on abduction - Supraspinatus weakness - Weakness on external rotation - Positive drop arm test   **Biceps**   - Pain on resisted flexion of the elbow or resisted supination of the forearm - Positive Speeds test   Additional tests:  Lift off tests for subscapularis |
| **FLEXOR-EXTENSOR PERITENDINITIS OR TENOSYNOVITIS OF THE FOREARM – WRIST REGION**  Inflammation of the extensor or flexor tendons of wrist | - Pain of the ventral or dorsal aspect of the hand, wrist or forearm - Pain on wrist movement localised to the affected tendon sheaths in the wrist - Symptoms present now or on at least 4 days during the last 7 days   Additional features:   - History of crepitus, swelling or tenderness of tendon sheath - Triggering/locking/nodule on tendon - Absence of pain in the neck or neurological symptoms in the median nerve distribution | - Reproduction of pain by resisted movement of the affected tendons with the forearm stabilised - AND Pain on palpation of the affected tendons - OR palpable crepitus under the symptom area - OR visible swelling of the dorsum of the wrist-forearm   Additional signs:   - Pain on active wrist flexion/extension - Pain on passive stretch of tendons) |
| **DE QUERVAINS**  Painful swelling of the first compartment containing extensor pollicis brevis and abductor pollicis longus | - At least intermittent pain which is centred over the radial styloid - exacerbated by resisted thumb extension - Symptoms present now or on at least 4 days during the last 7 days   Additional features:   - Pain can radiate into the hand and forearm | - AND tender swelling of the first extensor compartment - AND either pain reproduced by resisted thumb extension - OR resisted thumb abduction - OR positive Finkelstein’s test   Additional features:   - Pain worsened by abduction or extension of the thumb |
| **EPICONDYLITIS – LATERAL AND MEDIAL**  Lateral : A lesion at the common extensor origin of the lateral epicondyle of the humerus  Medial: A lesion of the common flexor origin of the medial epicondyle of the humerus | ***Lateral Epicondylitis***   - At least intermittent activity-dependent lateral epicondylar pain - Symptoms present now or on at least 4 days during the last 7 days - Pain associated with active and resisted movements of the extensor muscles of the forearm   Additional Features:   - Absence of shoulder pain   ***Medial Epicondylitis***   - At least intermittent activity-related medial epicondylar pain - Symptoms present now or on at least 4 days during the last 7 days - Pain or burning emanating from the medial aspect if the elbow - Medial elbow pain exacerbated by repetitive wrist movements | ***Lateral Epicondylitis***   - Localised epicondylar pain on resisted extension of the wrist - Tenderness on palpation of the lateral epicondylar region   ***Medial Epicondylitis***   - Localised medial epicondylar pain on resisted flexion of the wrist - Tenderness on palpation over the medial epicondyle |
| **SHOULDER CAPSULITIS**  A condition characterised by the current or past pain in the upper arm with global restriction of glenohumeral movement in a capsular pattern | History of unilateral pain in the deltoid area | Equal restriction of active and passive GH movement in a capsular pattern i.e. external rotation > abduction > internal rotation |
| **SUBACROMIAL IMPINGEMENT** | - At least intermittent pain in the shoulder/deltoid region without paraesthesia ; pain worsened by active elevation movement of the upper arm - Symptoms present now or on 4 days during the last 7 days | A Positive findings in at least 5 of the following tests:   - Hawkins-Kennedy test - Neer’s test - Horizontal Adduction test - Speed’s test - Yergason’s test - Painful arc test |
| **THORACIC OUTLET SYNDROME** | A constellation of signs and symptoms in the arm or hand caused by compression of the neurovascular bundle at the thoracic outlet | Five of the following positive:   - Adson’s test (reproduction of symptoms) - Wright’s test- reproduction of symptoms - Wright’s tests -radial pulse abolished - Hyperabduction test – radial pulse abolished - Roo’s test - Tinel’s test |
| **ARTHRITIS OF THE UPPER EXTREMITY** | - Intermittent pain, locally or present around the joint - OR local stiffness after a period of rest or specific movement related cause - Symptoms present now or on at least 4 days of the last 7 days - Early morning stiffness and joint pain or swelling | - Capsular pattern in restricted passive movement of the joint |
| **RADIATING NECK PAIN** | - At least intermittent pain or stiffness in the neck - AND pain or paraesthesia in ≥1 upper extremity region in associated with head movements | - Pain in the upper extremity on active or passive cervical rotation   Other tests not mentioned   - Positive Spurlings test for radiculopathy - Significant levels of cervical limitation |
| **RAYNAUDS PHENOMENON/ HAND-ARM VIBRATION SYNDROME** | - Pain or paraesthesia in the digits - At least occasional attacks of well demarcated blanching in at least 1 of the digits - Attack triggered by exposure to environmental cold - AND a history of exposure to hand-arm vibration preceding symptoms - Symptoms present now or on at least 4 days of the last 7 days - Trophic skin changes and cyanotic colour in hand or digits | - Blanching of at least one fingertip; blanching observed or provoked by cold water test - Positive sensory tests (light touch, pain, temperature) AND positive 2 point discrimination test - Tenderness or swelling if the digits, hand and forearm - Muscle weakness if the hand - Joint pain and arthroses of the hand, wrist, elbow, neck etc |
| **FIBROMYALGIA** | - Generalised pain in association with pain over shoulders, trunk and usually thigh and tender point counts - Compulsory history of widespread pain=> pain both sides of body, pain above and below the waist, axial skeletal pain.   Additional Features   - Pain and neurological manifestations - Neurocognitive dysfunction - Fatigue - Sleep dysfunction - Autonomic and/or neuroendocrine dysfunction | - Compulsory pain on palpation of 11 or more of the 18 defined tender points |
